# The Influence of pH on Phosphatidylethanolamine Monolayer at the Air/Aqueous Solution Interface

**DOI:** 10.1007/s12013-012-9424-4

**Published:** 2012-09-09

**Authors:** Aneta Dorota Petelska, Monika Naumowicz, Zbigniew Artur Figaszewski

**Affiliations:** 1 Institute of Chemistry, University in Bialystok, Al. J. Pilsudskiego 11/4, 15-443 Bialystok, Poland; 2 Laboratory of Electrochemical Power Sources, Faculty of Chemistry, University of Warsaw, Pasteur St. 1, 02-093 Warsaw, Poland

**Keywords:** Monolayer, Phosphatidylethanolamine, Interfacial tension, pH, acid–base equilibria

## Abstract

The dependence of the interfacial tension of a phosphatidylethanolamine (PE) monolayer on the pH of the aqueous solution has been studied. A theoretical equation is derived to describe this dependence. A simple model of the influence of pH on the phosphatidylethanolamine monolayer at the air/hydrophobic chains of PE is presented. The contributions of additive phosphatidylethanolamine forms (both interfacial tension values and molecular area values) depend on pH. The interfacial tension values and the molecular area values for PEH^+^ and PEOH^−^ forms of phosphatidylethanolamine were calculated. The assumed model was verified experimentally. The experimental results agreed with those derived from the theoretical equation in a whole range of pH values.

## Introduction

Lipid Langmuir monolayers are considered a simple but a very efficient model of biological membranes and frequently applied in physics, chemistry, and biomedical sciences. With this technique, it is possible to obtain homogenous distribution of the phospholipid molecules in two-dimensional space, which is the water/air interface. The study of monolayers is of crucial importance in a great number of processes, including cell membrane modeling [1, 2], breathing mechanics [3, 4], vesicle formation [5, 6], and optical and electronic device fabrication [7, 8]. Monolayer systems are often characterized by their surface pressure–area (*π*–*A*) isotherms, which provide useful information concerning molecular level interactions between the components [9–11].

Phospholipids are major fractions of lipids found in biological membranes. Since monolayers—especially at the air/water interface—are commonly used as simplified models of biomembrane, many studies have been concentrated on them [12, 13].

Phosphatidylethanolamine is one of the most abundant lipids in eukaryotic cell membranes unevenly distributed between the inner and the outer leaflets of the bilayer. The higher ratio of PEs in the membrane leaflet facing the inner media in comparison to the external one has called the attention to the topological properties of those surfaces with the expectation that they may be a key to functional roles of this lipid [14–17].

Phosphatidylethanolamine is a neutral, zwitterionic phospholipid with an amphiphilic character. The surface pressure–area per molecule (π–*A*) curves of phosphatidylethanolamine have been reported previously [18–22]. Phillips and Chapman [22] obtained surface pressure–area data for the homologous series of saturated 1,2-diacyl phosphatidylcholines and phosphatidylethanolamines at the air–water interface. The results are compared with data already in the literature and the various physical states of the monolayers are described. The phosphatidylcholines formed more expanded films than the phosphatidylethanolamines and this is interpreted in terms of differences in the size and orientation of the polar groups. The heats and entropies associated with the transition from condensed to liquid-expanded film were calculated for dipalmitoylcholine. The values of these thermodynamic parameters were similar to those observed for the transition from gel to smectic mesophase for this phospholipid. This transition occurring in the bimolecular lamellae in water corresponds to the transition from condensed to expanded monolayer [22].

The pH dependence of amphiphilic substances at the air–water interface was investigated even earlier, at the beginning of the last century [23, 24]. Not only the interfacial tension values were recorded but also the ionic properties of a monolayer were studied by means of investigation of its surface potential at a fixed value of area/molecule.

While a phosphatidylethanolamine monolayer is not altered by the pH of subphase in some pH regions, a recent study connected with bilayer has shown that there is a maximum of interfacial tension at a certain pH [25]. A careful study is thus pertinent. Since the changes in interfacial tension values induce the changes of the values in the area per molecule, it is very important, in context of biological membranes, to know the exact molecular packing in various pH solutions.

This paper is a continuation of studies of the effect of pH on phospholipid monolayer at the air/aqueous solution interface. In this work, membranes have been formed from phosphatidylethanolamine. PE molecule is electrically neutral lipid, because it has two electrostatic charges, one negative (phosphate group) and one positive (ethanolamine group), on the specific locations in the hydrophilic head. The phosphatidylethanolamine molecule forms ampholyte ions and can participate in equilibrium with ions H^+^ as well as with OH^−^. Using the derived equations, we present a model of ion–monolayer interaction based on the calculations employing π–*A* curves.

## Theory

Since the phosphatidylethanolamine molecule (PE) possesses a zwitterionic character, it can participate in equilibrium reactions with both hydrogen ions and hydroxyl anions.

1 $$ {\text{PE}} + {\text{H}}^{ + } \Leftrightarrow {\text{PEH}}^{ + } $$

2 $$ {\text{PE}} + {\text{OH}}^{ - } \Leftrightarrow {\text{PEOH}}^{ - } $$

3 $$ {\text{PE}} + {\text{HOH}} \Leftrightarrow {\text{PEHOH}} $$

Consequently, equations of associations Eqs. (1, 2, 3) can be considering as the description of an adsorption process. As a result of adsorption of H^+^ and OH^−^ ions on the surface of phosphatidylethanolamine layer, the PE molecule can exist in four different forms. We shall consider the following forms: PEH^+^ with H^+^ adsorbed, PEOH^−^ with OH^−^ adsorbed, PEHOH with both H^+^ and OH^−^ ions adsorbed on the surface, and a free phosphatidylethanolamine molecule PE i.e., with no ions adsorbed. A phosphatidylethanolamine monolayer is assumed to consist of these four forms. The relative contributions of above forms are dependent on pH, according to Eqs. (1, 2, 3).

One can write three equations for equilibrium Eqs. (1, 2, 3) containing the equilibrium constants of these equilibria. On the basis of these equations, the activity of following phosphatidylethanolamine forms can be calculated [13]:

4 $$ a_{{{\text{PEH}}^{ + } }} = K_{{{\text{PEH}}^{ + } }} a_{\text{PE}} a_{{{\text{H}}^{ + } }} $$

5 $$ a_{{{\text{PEOH}}^{ - } }} = K_{{{\text{PEOH}}^{ - } }} a_{\text{PE}} a_{{{\text{OH}}^{ - } }} $$

6 $$ a_{\text{PEHOH}} = K_{\text{PEHOH}} a_{\text{PE}} $$

where $a_{\text{PE}} ,a_{{{\text{PEH}}^{ + } }} ,a_{{{\text{PEOH}}^{ - } }} ,a_{\text{PEHOH}}$ is the surface concentration of PE, PEH^+^, PEOH^−^, and PEHOH form of phosphatidylethanolamine (mol m^−2^); $a_{{{\text{H}}^{ + } }} ,a_{{{\text{OH}}^{ - } }}$ are the concentrations of ions in the subphase (mol m^−3^); $K_{{{\text{PEH}}^{ + } }} ,K_{{{\text{PEOH}}^{ - } }} ,K_{\text{PEHOH}}$ are the equilibrium constants of adsorption process of H^+^ or OH^−^ ions on phosphatidylethanolamine (m^3^ mol^−1^).

The sum of surface concentrations of any phosphatidylethanolamine forms at the air/water interface has to be equal to total surface concentrations of phosphatidylethanolamine (*s*). This *s* concentration can be easily measured using the π–*A* isotherms.

Moreover, the sum of the area fractions of these four phosphatidylethanolamine forms should give unity.

These relationships are described by following equations:

7 $$ a_{\text{PE}} + a_{{{\text{PEH}}^{ + } }} + a_{\text{PEHOH}} + a_{{{\text{PEOH}}^{ - } }} = s $$

8 $$ a_{\text{PE}} A_{\text{PE}} + a_{{{\text{PEH}}^{ + } }} A_{{{\text{PEH}}^{ + } }} + a_{\text{PEHOH}} A_{\text{PEHOH}} + a_{{{\text{PEOH}}^{ - } }} A_{{{\text{PEOH}}^{ - } }} = 1 $$

where *s*, total surface concentration of phosphatidylethanolamine measured by π–*A* isotherms (mol m^−2^); $A_{\text{PE}} ,A_{{{\text{PEH}}^{ + } }} ,A_{{{\text{PEOH}}^{ - } }} ,A_{\text{PEHOH}}$, area occupied by one mole of components PE, PEH^+^, PEOH^−^, and PEHOH (Å^2^ molec.^−1^).

The Eqs. (4, 5, 6, 7, 8) describe quantitatively the model of the influence of pH subphase on a phosphatidylethanolamine monolayer. The different forms of phosphatidylethanolamine would give monolayers, built from one component, that have different stability constant. The value of surface concentrations of any phosphatidylethanolamine forms affects the molecular packing of the head groups, which—in a consequence—influences the interfacial tension of lipid monolayer. Depending on the pH of subphase, the surface concentrations will change as the area per molecule changes. Different forms of phosphatidylethanolamine will have different areas per molecules depending on the contribution of the forms to the total amount of phosphatidylethanolamine molecules.

After elimination of $a_{\text{PE}}$, $a_{{{\text{PEH}}^{ + } }}$, $a_{\text{PEHOH}}$ and $a_{{{\text{PEOH}}^{ - } }}$ terms from the Eqs. (4, 5, 6, 7, 8), one obtains [13]:

9 $$ \frac{1}{s} = \frac{{A_{1} + a_{{{\text{H}}^{ + } }} A_{{{\text{PEH}}^{ + } }} K_{{{\text{PEH}}^{ + } }} + a_{{{\text{OH}}^{ - } }} A_{{{\text{PEOH}}^{ - } }} K_{{{\text{PEOH}}^{ - } }} }}{{A_{2} + a_{{{\text{H}}^{ + } }} K_{{{\text{PEH}}^{ + } }} + a_{{{\text{OH}}^{ - } }} K_{{{\text{PEOH}}^{ - } }} }} $$

where:

$$ A_{1} = A_{\text{PE}} + A_{\text{PEHOH}} K_{\text{PEHOH}} $$

$$ A_{2} = K_{\text{PEHOH}} + 1 $$

The direct form of the Eq. (9) is not convenient for calculations. After substituting the concentration of OH^−^ ions by the quotient of $K_{{{\text{H}}_{2} {\text{O}}}}$ and H^+^ concentration, one can divide the numerator of the above polynomial by its denominator. As a result, we obtain the series of terms containing the decreasing powers of H^+^ ions concentration. The equation obtained by multiplication by $a_{{{\text{H}}^{ + } }}$ is in the form where one can treat the negative terms as negligible. In consequence, such equation would have the linear character.

For large H^+^ concentrations, i.e., when $a_{{{\text{H}}^{ + } }} \to \infty$; the Eq. (9) will assume the following form [13]:

10 $$ \frac{{a_{{{\text{H}}^{ + } }} }}{s} = A_{{{\text{PEH}}^{ + } }} a_{{{\text{H}}^{ + } }} + \frac{{A_{1} - A_{2} A_{{{\text{PEH}}^{ + } }} }}{{K_{{{\text{PEH}}^{ + } }} }} $$

Equation (9) can be treated in the analogous way after substitution of H^+^ ion concentrations by concentrations of hydroxyl ions.

For large OH^−^ concentrations i.e., when $a_{{{\text{OH}}^{ - } }} \to \infty$; one can obtain [13]:

11 $$ \frac{{a_{{{\text{OH}}^{ - } }} }}{s} = A_{{{\text{PEOH}}^{ - } }} a_{{{\text{OH}}^{ - } }} + \frac{{A_{1} - A_{2} A_{{{\text{PEOH}}^{ - } }} }}{{K_{{{\text{PEOH}}^{ - } }} }} $$

Using these latter relationships, one can easily calculate the values of $A_{{{\text{PEH}}^{ + } }}$ and $A_{{{\text{PEOH}}^{ - } }}$ by regression in the region of large H^+^ and OH^−^ concentration values, respectively.

The next Eq. (12) can be used for verification of the calculated values against the experimental ones obtained on the basis of Eqs. (10) and (11).

Good agreement between them will mean that the system is well described by the above equations.

In order to verify this agreement, the Eq. (9) should be presented in the following form [13]:

12 $$ \frac{1}{s} = \frac{{\frac{{A_{1} }}{{K_{{{\text{PEOH}}^{ - } }} }} + a_{{{\text{H}}^{ + } }} A_{{{\text{PEH}}^{ + } }} \frac{{K_{{{\text{PEH}}^{ + } }} }}{{K_{{{\text{PEOH}}^{ - } }} }} + a_{{{\text{OH}}^{ - } }} A_{{{\text{PEOH}}^{ - } }} }}{{\frac{{A_{2} }}{{K_{{{\text{PEOH}}^{ - } }} }} + a_{{{\text{H}}^{ + } }} \frac{{K_{{{\text{PEH}}^{ + } }} }}{{K_{{{\text{PEOH}}^{ - } }} }} + a_{{{\text{OH}}^{ - } }} }} $$

$\frac{{K_{{{\text{PEH}}^{ + } }} }}{{K_{{{\text{PEOH}}^{ - } }} }}$ value is required for further calculations.

The equation needed to calculate this expression can be obtained from Eqs. (4) and (5). Its value can be calculated using the values of $a_{{{\text{PEH}}^{ + } }}$ and $a_{{{\text{PEOH}}^{ - } }}$ at the isoelectric point.

On the basis of the assumed model the interfacial tension can be calculated, provided that the interfacial tension value of phosphatidylethanolamine layer is the sum of the contributions from all forms i.e., ideal mixing of the different forms of phosphatidylethanolamine.

As was mentioned above, the values of the molecular area of phosphatidylethanolamine influence the interfacial tension values of the relative phosphatidylethanolamine forms.

The surface concentrations of phosphatidylethanolamine forms are the same as described by Eqs. (4, 5, 6). The Eqs. (13) and (14) describe further dependencies in the studied system.

13 $$ A_{i} = \frac{{\gamma_{i}^{0} }}{\gamma s} $$

14 $$ \gamma = \gamma_{{{\text{PEH}}^{ + } }}^{0} + \gamma_{\text{PEHOH}}^{0} + \gamma_{\text{PE}}^{0} + \gamma_{{{\text{PEOH}}^{ - } }}^{0} $$

where *A*_*i*_ is the area occupied by one mole of adequate form of phosphatidylethanolamine (PE, PEH^+^, PEOH^−^, and PEHOH (Å^2^ molec.^−1^), $\gamma_{i}^{0}$ is the interfacial tension of the adequate form of phosphatidylethanolamine (mN m^−1^); $\gamma$ is the measured interfacial tension obtained from the π–*A* isotherms.

As the interfacial tension can be treated as the interfacial energy concentrated at the interfaces, we assume—based on the additivity rule—that the interfacial tension of the phosphatidylethanolamine layer is a sum of the interfacial tensions values of the PE forms.

Then, the relationship between the surface concentration, the total surface concentration *s*, and the interfacial tension value is obtained:

15 $$ \gamma = \gamma_{\text{PEHOH}}^{0} \frac{{a_{\text{PEHOH}} }}{s} + \gamma_{{{\text{PEH}}^{ + } }}^{0} \frac{{a_{{{\text{PEH}}^{ + } }} }}{s} + \gamma_{{{\text{PEOH}}^{ - } }}^{0} \frac{{a_{{{\text{PEOH}}^{ - } }} }}{s} + \gamma_{\text{PE}}^{0} \frac{{a_{\text{PE}} }}{s} $$

After the substitution of Eqs. (4, 5, 6) into Eq. (15) we obtain [13]:

16 $$ \gamma = \frac{{\gamma_{1} + a_{{{\text{H}}^{ + } }} \gamma_{{{\text{PEH}}^{ + } }}^{0} K_{{{\text{PEH}}^{ + } }} + a_{{{\text{PEOH}}^{ - } }} \gamma_{{{\text{PEOH}}^{ - } }}^{0} K_{{{\text{PEOH}}^{ - } }} }}{{a_{{{\text{H}}^{ + } }} K_{{{\text{PEH}}^{ + } }} + a_{{{\text{OH}}^{ - } }} K_{{{\text{PEOH}}^{ - } }} }} $$

where

$$ \gamma_{1} = \gamma_{\text{PE}}^{0} + \gamma_{\text{PEHOH}}^{0} K_{\text{PEHOH}} $$

In analogy to the above equations describing the areas per molecules, the polynomial Eq. (16) and adequate approximations lead to the following forms depending on the conditions:

For large H^+^ concentrations, i.e., when $a_{{{\text{H}}^{ + } }} \to \infty$ [13];

17 $$ \gamma a_{{{\text{H}}^{ + } }} = \gamma_{{{\text{PEH}}^{ + } }}^{0} a_{{{\text{H}}^{ + } }} + \frac{{K_{\text{PEHOH}} (\gamma_{\text{PEHOH}}^{0} - \gamma_{{{\text{PEH}}^{ + } }}^{0} ) + (\gamma_{\text{PE}}^{0} - \gamma_{{{\text{PEH}}^{ + } }}^{0} )}}{{K_{{{\text{PEH}}^{ + } }} }} $$

This approximation enables the calculation of the interfacial tension value of phosphatidylethanolamine form with adsorbed H^+^ ions.

Analogously, for basic solutions, when $a_{{{\text{OH}}^{ - } }} \to \infty$ [13];

18 $$ \gamma a_{{{\text{OH}}^{ - } }} = \gamma_{{{\text{PEOH}}^{ - } }}^{0} a_{{{\text{OH}}^{ - } }} + \frac{{K_{\text{PEHOH}} (\gamma_{\text{PEHOH}}^{0} - \gamma_{{{\text{PEOH}}^{ - } }}^{0} ) + (\gamma_{\text{PE}}^{0} - \gamma_{{{\text{PEOH}}^{ - } }}^{0} )}}{{K_{{{\text{PEOH}}^{ - } }} }} $$

The accuracy of the assumed model—the additivity of the phosphatidylethanolamine forms—can be verified with the help of Eq. (19) [13].

19 $$ \gamma = \frac{{\frac{{\gamma_{1} }}{{K_{{{\text{PEOH}}^{ - } }} }} + a_{{{\text{H}}^{ + } }} \gamma_{{{\text{PEH}}^{ + } }}^{0} \frac{{K_{{{\text{PEH}}^{ + } }} }}{{K_{{{\text{PEOH}}^{ - } }} }} + a_{{{\text{PEOH}}^{ - } }} \gamma_{{{\text{PEOH}}^{ - } }}^{0} }}{{\frac{{\gamma_{2} }}{{K_{{{\text{PEOH}}^{ - } }} }} + a_{{{\text{H}}^{ + } }} \frac{{K_{{{\text{PEH}}^{ + } }} }}{{K_{{{\text{PEOH}}^{ - } }} }} + a_{{{\text{OH}}^{ - } }} }} $$

where

$$ \gamma_{1} = \gamma_{\text{PE}}^{0} + \gamma_{\text{PEHOH}}^{0} K_{\text{PEHOH}} $$

$$ \gamma_{2} = K_{\text{PEHOH}} + 1 $$

## Experimental

### Measuring Apparatus and Measuring Procedures

The homemade computer-controlled apparatus used for surface tension measurements was presented in previous paper [26].

The surface tension measurements were carried out at the water/air interface at 22 °C, and were expressed as surface pressure–area per molecule (π–*A*) isotherms. For all experiments, the trough was filled with triple-distilled water as the subphase. The monolayers were prepared by spreading a defined volume of a lipid solution in 1-chloropropane on the aqueous subphase using a Hamilton micro-syringe. Ten minutes were allowed for solvent evaporation and monolayer equilibration before an experiment was begun. The monolayer was continuously compressed to obtain the surface pressure–area per molecule (π–*A*) isotherms using the glass barrier (barrier was moved at a velocity of 0.03 cm s^−1^). The Nima ST9002 computer program was used to calculate the surface pressure (π) of the monolayer as a function of surface area per molecule (*A*): *π* = *γ* − *γ*_0_ = *f*(*A*), where *γ*_0_ is the surface tension of the lipid-covered surface and *γ* is the surface tension of the bare air/water interface [27].

Before each trial, the Teflon trough (trough size 648 cm^2^) was washed and rinsed with purified water. The subphase surface was cleaned just prior to each measurement by suction with a vacuum pump until the surface tension was constant and equal to the surface tension value of pure water at 22 °C (~72 mN m^−1^). All glassware in contact with the samples was cleaned with chromic acid and repeatedly rinsed with purified water before use.

The system was enclosed in an acrylic box to minimize water evaporation, to ensure high humidity, and to avoid contamination of the system.

All of the reported values are highly reproducible and represent the average of at least five experiments. The standard deviation for surface area measurements was <1 %.

### Reagents

Phosphatidylethanolamine (99 %) was purchased from Fluka and was used as received. The molecular weight of the phosphatidylethanolamine was ~752.08 g mol^−1^.

The 1-chloropropane solvent (>98 % pure) was supplied by Aldrich. Solutions were prepared by dissolving appropriate amounts of each material in 1-chloropropane at a concentration of 1 mg cm^−3^ and were stored at 4 °C. The water used in the experiments was prepared by triple distillation (the second distillation was performed over KMnO_4_ and KOH to remove organic impurities).

Buffers of 2–12 pH ranges were prepared according to Britton and Robinson [28] and used as electrolyte. They were prepared by adding 0.2 M sodium hydroxide to 100 ml of solution having the following composition: 0.04 M 80 % acetic acid produced by Polish Chemical Reagents (POCh), 0.04 M phosphoric acid from POCh, and 0.04 M boric acid from POCh. A suitable pH of the buffer was established depending on the amount of added sodium hydroxide. Initial pH of the prepared buffer is 1.81. It changes to e.g., 3.29 after 20 cm^3^ of NaOH from POCh was added or to 6.80 if 50 cm^3^ was added. Britton and Robinson buffer was used in the experiments because this solution is being applied to biochemical experiments as the standard buffer, because of the wide pH range (2–12) and because it does not interact with biological membranes.

## Results and Discussion

The measurements of interfacial tension values of lipid monolayer are useful for determination of the surface area per molecule. Dependence of some physical properties on pH is of interest for applications of biological membranes in biology and medical sciences. The dependence of the surface area per phosphatidylethanolamine molecule versus pH of the subphase could be obtained with the usage of the π–*A* isotherms.

**Fig. 1:**
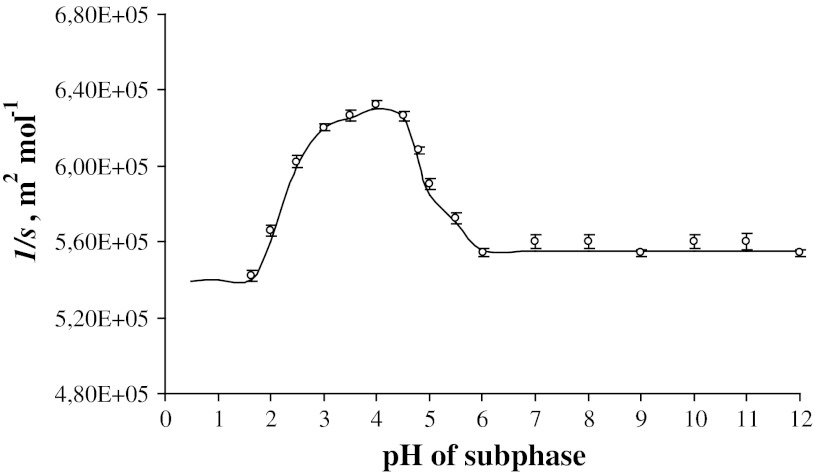
The inverse of surface concentration (1/*s*) of phosphatidylethanolamine as a function of pH subphase at surface pressure of ~30 mN m^−1^ (the experimental values are indicated by *points* and the theoretical values by the *curve*)

Figure 1 presents the measured values of the inverse of surface concentration of phosphatidylethanolamine as a function of pH subphase. The experimental values are denoted as points. The solid curve is calculated using the Eq. (12), as will be discussed later. When subphase is acidic (pH 2.0), the surface concentration *s* is equal to 1.84 × 10^−6^ mol m^−2^. Values of *s* increase steeply reaching a maximum close to the isoelectric point of phosphatidylethanolamine (surface concentration maximum value—6.32 × 10^5^ m^2^ mol^−1^ at pH 4.18). It is noteworthy that this maximum is obtained at the isoelectric point (4.18) of phosphatidylethanolamine, which was established for PE bilayer [25]. When pH of subphase increases further, the values of the phosphatidylethanolamine surface concentration decrease steeply within 4.18–6.00 pH range. The pH regions between 6 and 12, as can be seen from Fig. 1, are characterized by only small variations.

Employing on the assumed model, one can calculate the area of PEH^+^ form, using the Eq. (10). Its value is 5.45 × 10^5^ m^2^ mol^−1^ (90.5 Å^2^ molec.^−1^). However, since the extrapolation from only a few experimental points can produce unreliable results, we have proceeded differently. In order to confirm the obtained result, we calculated the value of PEH^+^ phosphatidylethanolamine form by fitting the experimental curve using the algorithm for least

square estimation of parameters. Then, it is equal to 6.28 × 10^5^ m^2^ mol^−1^ (104 Å^2^ molec.^−1^). It is noteworthy that we can use the extrapolated PEOH^−^ value of surface concentration for such calculations. From Fig. 1 one can see that in the pH range of 6–12, we can treat the experimental points as reliable. The respective surface concentration value of PEOH^−^ form is equal to 5.54 × 10^5^ m^2^ mol^−1^ (92 Å^2^ molec.^−1^).

**Fig. 2:**
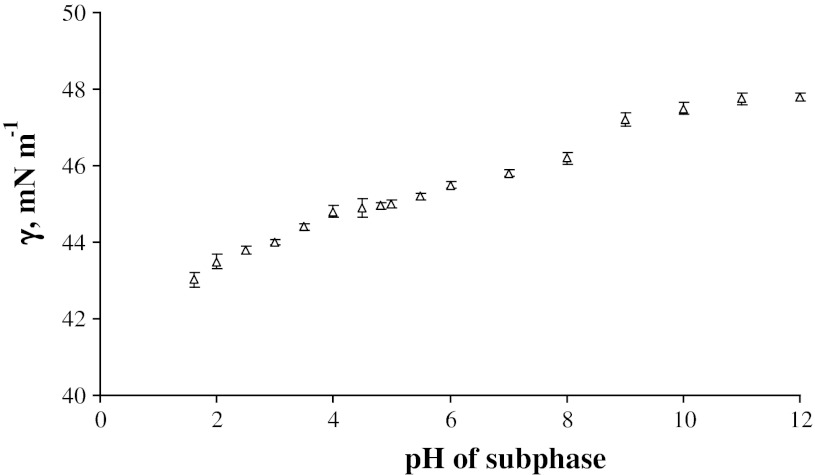
The interfacial tension (γ) values of the phosphatidylethanolamine monolayer at the air/aqueous solution versus pH of this solution

Figure 2 presents the interfacial tension values measured for phosphatidylethanolamine monolayer. As it can be seen, the values in the 2–6 pH range are only slightly changed with increasing pH of subphase. A further decrease in the H^+^ concentration results in an abrupt change of the plot. The interfacial tension values start to increase continuously up to pH 8–10. For large OH^−^ concentrations, the interfacial tension values are almost the same.

In the Langmuir approach [29], an air–hydrophobic layer interfacial tension value and polar layer-aqueous subphase interfacial tension value make up the monolayer surface tension. The interfacial tension values for the interface of the hydrophobic chains–hydrophobic chains in a phospholipid bilayer are assumed negligible. Moreover, the values of the interfacial tension at the interface of headgroups of phospholipid-subphase are the same in both cases: for a monolayer and for a bilayer. As a result, the difference between monolayer interfacial tension obtained experimentally and the interfacial tension of bilayer equals the interfacial tension of hydrophobic layer–air interface. We proceed in a similar way as Jähning [30], who approximated hydrophobic layer–air by the interfacial tension of n-alkane–air interface. Since the values of the interfacial tension of the hydrophobic chains–air interface are dominating, these values were applied to the system and used in further calculation [31].

**Fig. 3:**
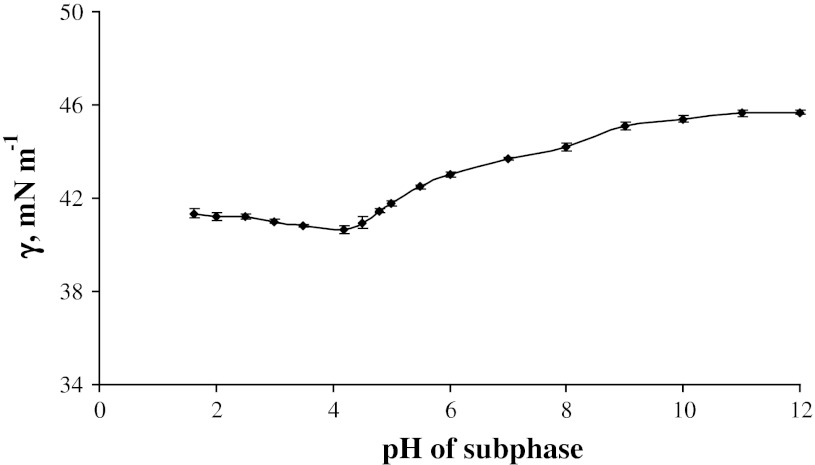
The interfacial tension (γ) of the phosphatidylethanolamine monolayer at the air/hydrophobic chains of examined phospholipid as a function of pH (the experimental values are indicated by *points* and the theoretical values by the *curve*)

In Fig. 3, points denote the calculated values of interfacial tension of lipid monolayer values for interface of the air–phosphatidylethanolamine hydrophobic chains. As it was written above, these values are calculated as a difference of the experimental values for monolayer and bilayer composed of the same phosphatidylethanolamine [25]. The calculated values obtained from Eq. (19) are denoted in the same figure by the solid line.

It is worth emphasizing that for the hydrophobic chains–air interface this run is dependent on pH. The latter interface is dominating as far as the values of the interfacial tension are concerned. When the pH value approaches the isoelectric point, we obtain the minimum of the interfacial tension values. It is equal 40.65 mN m^−1^ at pH 4.18. With the changes of subphase pH, the interfacial tension values increase until the pH reaches 9.

The interfacial tension value of phosphatidylethanolamine monolayer on the basic subphase is then approximately 45.6 mN m^−1^.

We proceed to calculate the interfacial tension values. Then, interfacial tension values of PEH^+^ and PEOH^−^ forms are: 43.97 and 47.61 mN m^−1^. The calculated values of interfacial tension for pH less than 2 are calculated on the basis of Eq. (17). The results are presented in Fig. 3. Remarkably, the experimental values are in good agreement with the calculated ones presented in figure as a solid line.

The verification of the assumed model is presented in the form of the Eq. (12) for the molecular areas of phosphatidylethanolamine forms and as Eq. (19) for the interfacial tension values. As can be seen from the Fig. 3, the obtained values (solid line) are very close to the experimental results represented by points. The good agreement between points and the solid lines (representing the calculated values) means that the proposed model is well derived and the obtained values are correct. The results confirm the existence of four phosphatidylethanolamine forms, no matter whether a phosphatidylethanolamine exists as a monolayer or a bilayer.

The phosphate (P^−^) end of the phosphatidylethanolamine head group is anchored at the air/water interface, while the hydrocarbon chains are driven toward air, thus the hydrophobic effects drive the methyl and methylene groups around the N^+^ charge toward the hydrocarbon. In order to reduce hydrocarbon–water contact, the polar head groups are packed closely. This restricts the freedom of the lipid chains and results in them exerting a lateral pressure on the surroundings. The area of hydrophilic heads of lipids determines the whole value of the area per molecule.

The surface charge is dependent on pH and thus loosing protons can easily modify it. As we can see, the influence of pH of subphase results in the molecular packing of the phosphatidylethanolamine headgroup, and—directly—on the area taken up by chains at the air–chains interface. One can see when comparing Figs. 2 and 3 that the interfacial tension values for air–water interface is no so strongly dependent on pH as it is in the case of air–phospholipid chains.

## Conclusions

The assumed model is based on the additivity of the interfacial tension values and molecular area values of phosphatidylethanolamine forms. The contribution of following phosphatidylethanolamine forms: PEH^+^, PE, PEHOH, and PEOH^−^ depends on pH of subphase.

The interfacial tension values and the molecular areas values for PEH^+^ and PEOH^−^ forms of phosphatidylethanolamine were calculated. These values are equal to 5.45 × 10^5^ m^2^ mol^−1^ (90.5 Å^2^ molec.^−1^), 5.54 × 10^5^ m^2^ mol^−1^ (92 Å^2^ molec.^−1^), and 43.97, 47.61 mN m^−1^, respectively.

The value of the molecular area of hydrophilic heads of phosphatidylethanolamine determines the surface concentration of phosphatidylethanolamine and, as the result, the interfacial tension values at the air/hydrophobic chains interface. The difference between monolayer interfacial tension obtained experimentally and the interfacial tension of bilayer equals the interfacial tension of hydrophobic layer–air interface. The assumed model agreed well with the experimental values.

The mathematically derived and experimentally confirmed results presented in this paper are of great importance for the interpretation of surface phenomena occurring in lipid monolayers. These results can help provide a better understanding of the physical and physicochemical properties of biological membranes, including ion adsorption on the membrane surface and interfacial tension. For example, the interfacial tension of a biological membrane determines its rigidity and therefore affects its stability. Interfacial tension is affected by factors such as pH or the presence of substances incorporated in the lipid bilayer, for example cholesterol, other lipids, fatty acids, amines, amino acids, or proteins. The method proposed in this paper and in earlier studies [1, 2, 13, 26, 27] may be used with success to determine lipid–lipid and lipid–ion equilibria in lipid monolayers.
